# Distribution of nuchal translucency in antenatal screening for Down's syndrome

**DOI:** 10.1258/jms.2010.009107

**Published:** 2010-03

**Authors:** J P Bestwick, W J Huttly, N J Wald

**Affiliations:** Wolfson Institute of Preventive Medicine, Barts and the London Queen Marys School of Medicine and Dentistry, Charterhouse Square, London EC1M 6BQ, UK; Wolfson Institute of Preventive Medicine, Barts and the London Queen Marys School of Medicine and Dentistry, Charterhouse Square, London EC1M 6BQ, UK; Wolfson Institute of Preventive Medicine, Barts and the London Queen Marys School of Medicine and Dentistry, Charterhouse Square, London EC1M 6BQ, UK

## Abstract

**Objective:**

To determine whether the standard deviation of nuchal translucency (NT) measurements has decreased over time and if so to revise the estimate and assess the effect of revising the estimate of the standard deviation on the performance of antenatal screening for Down's syndrome.

**Setting:**

Data from a routine antenatal screening programme for Down's syndrome comprising 106 affected and 22,640 unaffected pregnancies.

**Methods:**

NT measurements were converted into multiple of the median (MoM) values and standard deviations of log_10_ MoM values were calculated in affected and unaffected pregnancies. The screening performance of the Combined and Integrated tests (that include NT measurement) were compared using previous and revised estimates of the standard deviation.

**Results:**

The standard deviation of NT in unaffected pregnancies has reduced over time (from 1998 to 2008) (e.g. from 0.1329 to 0.1105 [log_10_ MoM] at 12–13 completed weeks of pregnancy, reducing the variance by about 30%). This was not observed in affected pregnancies. Compared with results from the serum, urine and ultrasound screening study (SURUSS), use of the revised NT standard deviations in unaffected pregnancies resulted in an approximate 20% decrease in the false-positive rate for a given detection rate; for example, from 2.1% to 1.7% (a 19% reduction) at a 90% detection rate using the Integrated test with first trimester markers measured at 11 completed weeks' gestation and from 4.4% to 3.5% (a 20% reduction) at an 85% detection rate using the Combined test at 11 completed weeks.

**Conclusions:**

The standard deviation of NT has declined over time and using the revised estimates improves the screening performance of tests that incorporate an NT measurement.

## INTRODUCTION

Nuchal translucency (NT) is useful as an antenatal screening marker for Down's syndrome in the late first trimester of pregnancy. It forms part of the Combined test (NT and serum markers pregnancy-associated plasma protein-A [PAPP-A] and free *β*-human chorionic gonadotrophin [free *β*-hCG] measured between 10 and 13 weeks gestation) and part of the Integrated test (NT and PAPP-A measured between 10 and 13 weeks and serum markers alphafetoprotein, unconjugated oestriol, free *β*-hCG and inhibin-A measured between 14 and 22 weeks gestation).

In monitoring our screening programme at the Wolfson Institute of Preventive Medicine, London, there was an indication that the standard deviation of NT in unaffected pregnancies decreased over time. This prompted us to investigate the observation further to obtain a revised estimate of the standard deviation. We then investigated the impact of the revised standard deviation on screening performance compared with results from the Serum, Urine and Ultrasound Screening Study (SURUSS).^1^

## METHODS

We used data from 22,719 women who attended two London antenatal clinics (at University College Hospital and the Whittington Hospital) between January 2003 and December 2008 for antenatal screening for Down's syndrome using the Combined or Integrated tests. The tests were performed at the Wolfson Institute of Preventive Medicine. We also included data from 27 women who were not offered these screening tests because of high NT measurements and whose pregnancies were subsequently diagnosed as being affected with Down's syndrome (median NT 4.9 mm). Down's syndrome pregnancies were recorded from the two hospitals, the regional cytogenetic unit and the National Down Syndrome Cytogenetic Register.

NT values were converted into multiple of the median (MoM) values by dividing measured NT values by the expected NT for a given crown rump length (CRL) (obtained from a log–linear regression of median log NT measurements against median CRL measurements in 5 mm categories of CRL [weighted by the number of women in each category]). Table 1 gives details of the pregnancies used in our analyses.

**Table 1:** Maternal and gestational age, and NT in Down's syndrome and unaffected pregnancies

	Down's syndrome	Unaffected
Median age (years)	39	34
Median CRL (mm)	63	62
**Gestational age**		
10 weeks gestation	2	243
11 weeks gestation	20	4270
12 weeks gestation	50	10,363
13 weeks gestation	34	7764
Total	106	22,640
Median NT (mm)	2.7	1.5
**Median NT (MoM)**		
10 weeks gestation	3.93	1.02
11 weeks gestation	2.22	0.98
12 weeks gestation	1.86	1.02
13 weeks gestation	1.55	0.99

CRL, crown rump length; NT, nuchal translucency, MoM, multiple of the median

Standard deviations of NT MoM values (based on all data) and the median NT MoM values according to gestational age were calculated in Down's syndrome and unaffected pregnancies. Gestational age was estimated from CRL using the equation reported by Robinson and Fleming.^2^

Screening performance was expressed as detection rates for specified false-positive rates, false-positive rates for specified detection rates and detection and false-positive rates for specified risk cut-offs. For the Combined and Integrated tests, screening performance was estimated for the population of maternities in England and Wales 1996–1998,^3^ for comparison with published estimates from SURUSS.^1^

Detection and false-positive rates were estimated by numerical integration of the multivariate Gaussian distributions of MoM values in Down's syndrome and unaffected pregnancies, using the maternal age distribution and distribution parameters (means, standard deviations and correlation coefficients) for serum markers previously published^1,4,5^ except for the standard deviation of NT in unaffected pregnancies, which was found to be significantly lower than estimates made in studies conducted in the past and the truncation limits for NT (see the Results). The median NT MoM values and standard deviations in Down's syndrome pregnancies were similar to those previously reported, so it was not necessary to revise these (see the Results). Screening performance estimates apply to the detection of Down's syndrome in the early second trimester. Too few data are available on affected pregnancies at 10 weeks, so screening performance was estimated for 11, 12 and 13 completed weeks of gestation only.

## RESULTS

Table 2 shows the expected median MoM values (regressed) and standard deviations of the (log_10_) MoM value in Down's syndrome and unaffected pregnancies in this study compared with those reported in SURUSS.^1^ The standard deviations in unaffected pregnancies were lower than those reported in SURUSS (the standard deviations at 12 and 13 weeks were similar [0.1096 and 0.1109, respectively], and therefore combined). In Down's syndrome pregnancies, the median MoM values and standard deviation were similar to those reported in SURUSS.

**Table 2:** Median, standard deviation and truncation limits of NT MoM values in Down's syndrome and unaffected pregnancies: estimates from the present study and estimates from SURUSS^1,4,5^

	Estimates from present study		Estimates from SURUSS	
	Down's syndrome pregnancies	Unaffected pregnancies	Down's syndrome pregnancies	Unaffected pregnancies
**Median NT MoM (regressed)**				
10 completed weeks	2.86	1	2.42	1
11 completed weeks	2.29	1	2.18	1
12 completed weeks	1.84	1	1.96	1
13 completed weeks	1.47	1	1.77	1
**Standard deviation (log_10_ MoM)**				
10 completed weeks	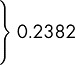	0.1550*	} 0.2313	0.1732*
11 completed weeks		0.1275^†^		0.1439^†^
12 completed weeks		0.1105^†^		0.1329^†^
13 completed weeks				
**Trunction limits (MoM)**				
10 completed weeks	0.50–2.50	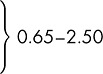		
11 completed weeks	0.70–2.50			
12 completed weeks	0.80–2.50			
13 completed weeks	0.85–2.50			

NT, nuchal translucency; SURUSS, Serum, Urine and Ultrasound Screening Study; MoM, multiple of the median

*P = 0.024

^†^*P* < 0.001

**Figure 1:**
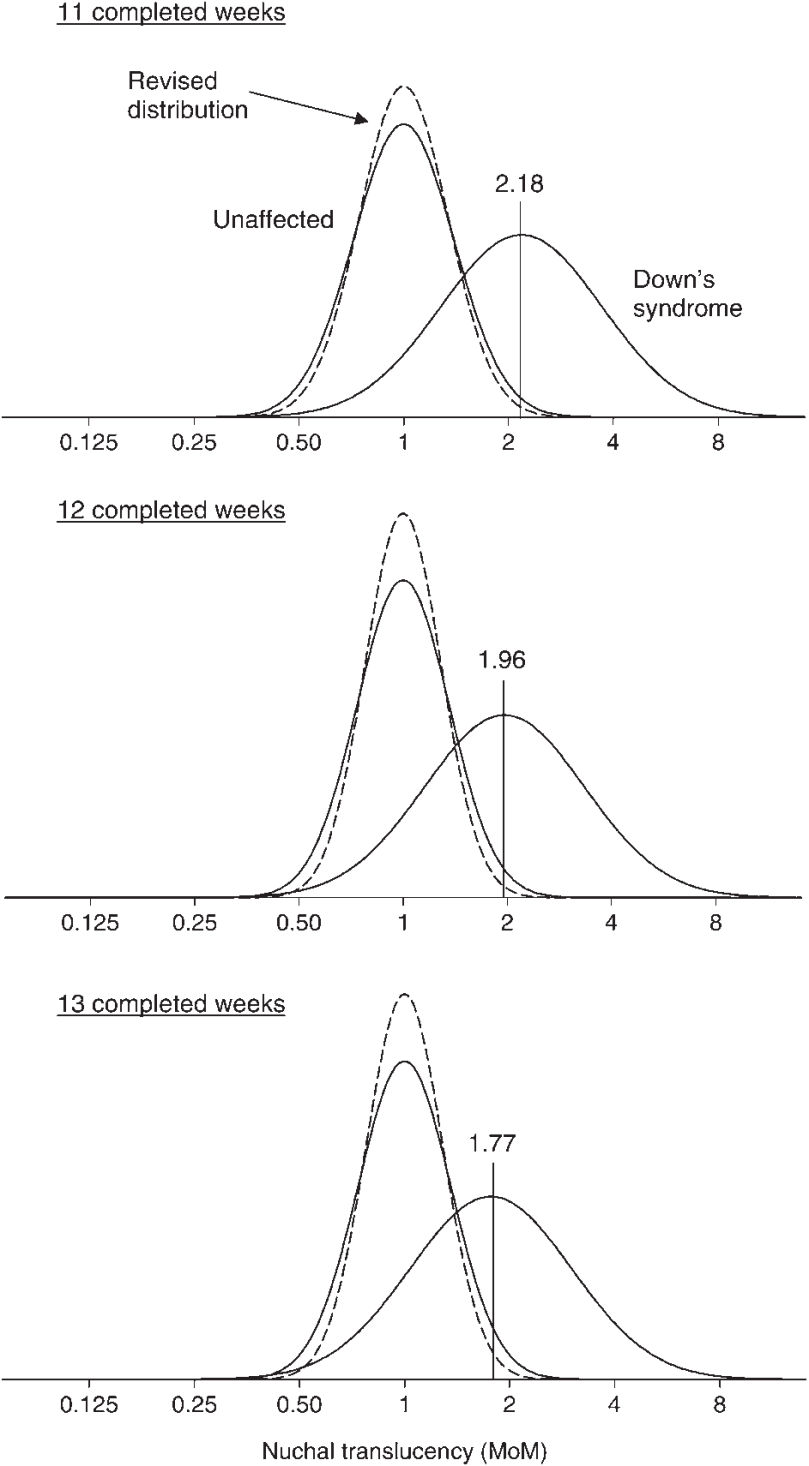
Relative frequency distributions of nuchal translucency (NT) multiple of the median (MoM) values in Down's syndrome and unaffected pregnancies according to gestational age. Solid line is previous distribution and dashed line is revised distribution. Median MoM in Down's syndrome pregnancies at vertical line

**Figure 2:**
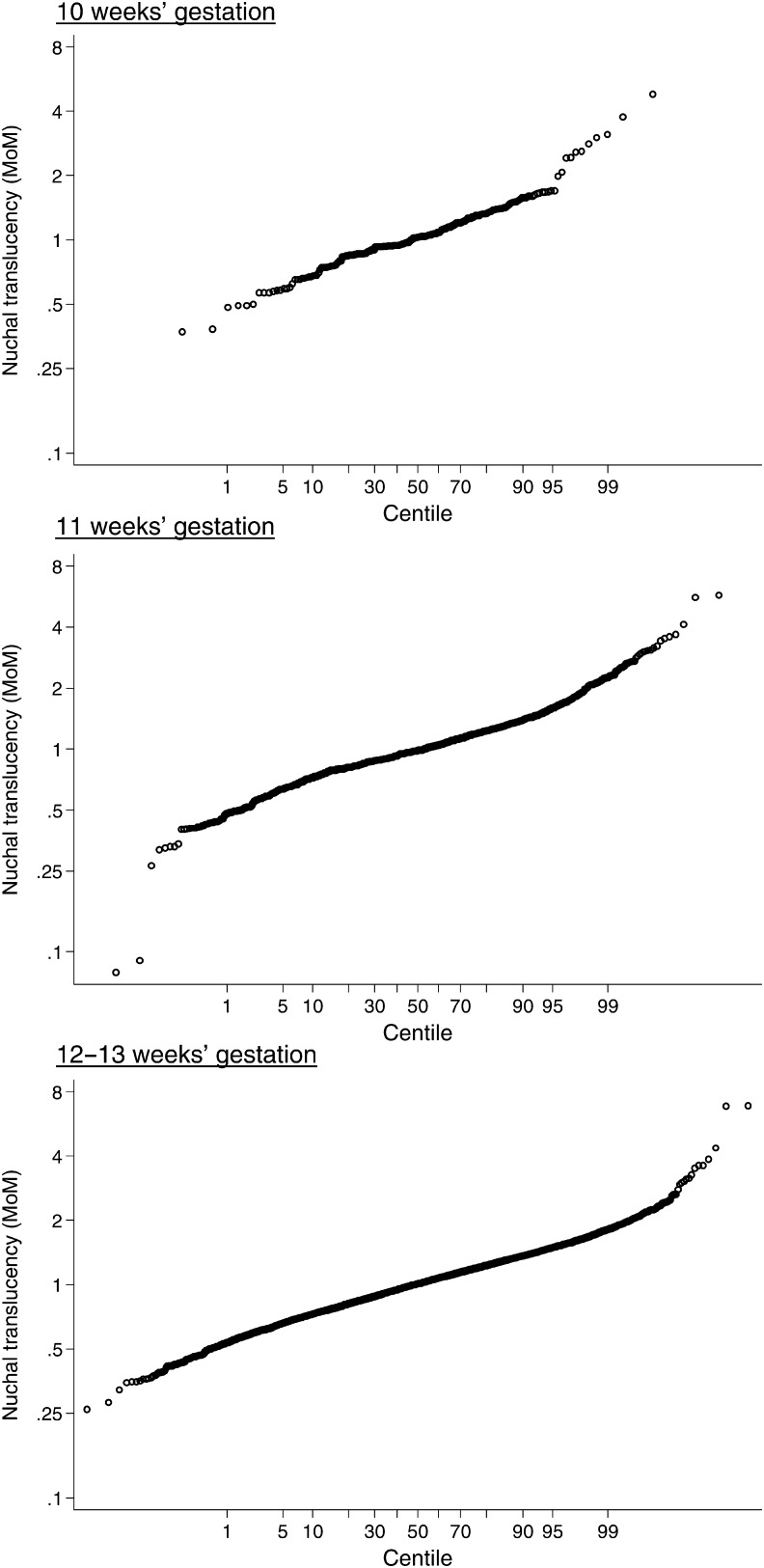
Probability plots of nuchal translucency in unaffected pregnancies according to week of gestation

Figure 1 shows the relative frequency distributions of NT in Down's syndrome and unaffected pregnancies using previous estimates of the standard deviation in unaffected pregnancies and revised estimates at 11, 12 and 13 completed weeks' gestation. As a single screening test considered without maternal age, NT alone has revised detection rates of 71%, 68% and 61% for a 5% false-positive rate at 11, 12 and 13 completed weeks, respectively, compared with previous estimates of 67%, 63% and 55%, respectively.^1^ Truncation limits for NT (also given in Table 2) were revised based on probability plots of NT MoM values (see Figure 2) and to prevent the reversal of risk at lower NT MoM values^4^ (i.e. risk of Down's syndrome decreasing with smaller NT measurements and then increasing with further decreases in NT).

Table 3 shows the effect of the revised distribution parameters based on the decreased NT standard deviation in unaffected pregnancies and the revised truncation limits compared with those from SURUSS on the screening performance of the Combined test at 11, 12 and 13 completed weeks' gestation and the Integrated test with first-stage measurements at 11, 12 and 13 completed weeks' gestation.

**Table 3:** Screening performance estimates of NT, Combined test and Integrated test according to completed week of first trimester measurements: estimates with revised standard deviations and truncation limits and estimates using SURUSS parameters

Screening test (include maternal age)	Estimates using revised unaffected standard deviations and revised truncation limits							Estimates using SURUSS parameters^1,4,5^						
	DR (%) for FPR of			FPR (%) for DR of				DR (%) for FPR of			FPR (%) for DR of			
	1%	3%	5%	75%	80%	85%	90%	1%	3%	5%	75%	80%	85%	90%
**Combined test**														
11 completed weeks	76	84	87	0.94	1.8	3.5	7.4	72	82	86	1.4	2.4	4.4	8.5
12 completed weeks	74	82	86	1.2	2.3	4.5	9.3	69	79	84	1.9	3.3	6.0	11
13 completed weeks	69	78	83	2.0	3.6	6.4	12	64	76	81	2.8	4.6	7.7	14
**Integrated test**														
11 completed weeks	87	93	95	0.13	0.27	0.64	1.7	86	92	94	0.20	0.41	0.87	2.1
12 completed weeks	86	92	94	0.16	0.37	0.88	2.3	83	90	93	0.30	0.61	1.3	3.0
13 completed weeks	82	89	92	0.35	0.73	1.6	3.6	80	88	91	0.55	1.1	2.1	4.4

SURUSS, Serum, Urine and Ultrasound Screening Study; NT, nuchal translucency; DR, detection rate; FPR, false-positive rate

The revised false-positive rates for a given detection rate are about 20% lower than previous estimates. For example, at an 85% detection rate the false-positive rate using the Combined test at 11 completed weeks' gestation is 3.5% instead of 4.4% (or about 9 fewer false-positives per 1000 women screened), and at a 90% detection rate using the Integrated test with first-stage measurements at 11 completed weeks' gestation, the false-positive rate is 1.7% instead of 2.1% (4 fewer false-positives per 1000 women screened). The relative reduction in the false-positive rate is greater at lower detection rates; for example, at an 80% detection rate the false-positive rate is reduced by 25% (from 2.4% to 1.8%) with the Combined test at 11 completed weeks.

Table 4 shows screening performance of the Combined and Integrated tests using the revised and the previous estimates according to risk cut-off.

**Table 4:** Screening performance estimates of the Combined test and Integrated test according to completed week of first trimester measurements and early second trimester risk cut-off: estimates with revised standard deviations and truncation limits and estimates using SURUSS parameters

Screening test (include maternal age)	Risk cut-off																	
	1 in 50			1 in 100			1 in 150			1 in 200			1 in 250			1 in 300		
	DR (%)	FPR (%)	OAPR	DR (%)	FPR (%)	OAPR	DR (%)	FPR (%)	OAPR	DR (%)	FPR (%)	OAPR	DR (%)	FPR (%)	OAPR	DR (%)	FPR (%)	OAPR
**Estimates using revised unaffected standard deviations and revised truncation limits**																		
Combined test																		
11 completed weeks	74	0.79	1:5	79	1.6	1:9	82	2.4	1:13	84	3.1	1:16	86	3.8	1:20	87	4.5	1:23
12 completed weeks	72	0.78	1:5	77	1.6	1:9	80	2.4	1:13	82	3.2	1:17	84	3.9	1:21	85	4.6	1:24
13 completed weeks	68	0.87	1:6	74	1.8	1:11	78	2.7	1:16	80	3.6	1:20	82	4.5	1:24	83	5.3	1:28
Integrated test																		
11 completed weeks	84	0.56	1:3	88	1.1	1:5	90	1.5	1:7	91	1.9	1:10	92	2.3	1:11	92	2.7	1:13
12 completed weeks	83	0.58	1:3	86	1.1	1:6	88	1.6	1:8	90	2.1	1:10	90	2.5	1:12	91	3.0	1:14
13 completed weeks	80	0.68	1:4	84	1.3	1:7	86	1.9	1:10	88	2.5	1:12	89	3.0	1:15	90	3.5	1:17
**Estimates using SURUSS parameters^1,4,5^**																		
Combined test																		
11 completed weeks	71	0.87	1:5	77	1.8	1:10	81	2.6	1:14	83	3.4	1:18	85	4.2	1:22	86	4.9	1:25
12 completed weeks	68	0.92	1:6	75	1.9	1:11	79	2.8	1:16	81	3.7	1:20	83	4.6	1:25	84	5.4	1:28
13 completed weeks	64	1.0	1:7	72	2.1	1:13	76	3.2	1:19	79	4.2	1:24	81	5.3	1:29	83	6.2	1:33
Integrated test																		
11 completed weeks	83	0.60	1:3	87	1.2	1:6	89	1.7	1:8	90	2.1	1:10	91	2.6	1:13	92	3.0	1:14
12 completed weeks	81	0.67	1:4	85	1.3	1:7	87	1.9	1:9	89	2.4	1:12	90	2.9	1:14	91	3.4	1:17
13 completed weeks	78	0.77	1:4	83	1.5	1:8	85	2.2	1:11	87	2.8	1:14	88	3.4	1:17	89	4.0	1:20

DR, detection rate; FPR, false-positive rate; OAPR, odds of being affected given a positive test result; SURUSS, Serum, Urine and Ultrasound Screening Study

## DISCUSSION

The standard deviation of NT in unaffected pregnancies has decreased over time. At 12–13 weeks gestation, our revised standard deviation of log_10_ NT MoM (0.1105) represents a 17% decrease in the standard deviation or a 31% decrease in the variance compared with the standard deviation reported in 2003 in SURUSS (0.1329).^1^ Data from the Fetal Medicine Foundation show a similar trend; an estimate of the standard deviation in unaffected pregnancies in 1998 was 0.120,^6^ compared with 0.097 in 2008 (from Figure 4 of Wright *et al.*^7^), a similar decrease in the standard deviation of 19% or decrease in the variance of 35%. The explanation for this decrease is uncertain; it could be a result of greater magnification of the fetal image with improvements in instrumentation together with sonographers having become more experienced in the measurement of NT.

A declining trend in the standard deviation in Down's syndrome pregnancies was not observed, perhaps because NT tends to be large in such pregnancies, so precise measurements would have been possible anyway. There were too few data to examine whether the standard deviation decreased with gestation as it did in unaffected pregnancies. Exclusion of data from women in whom clinical action was taken on grounds of a high NT alone results in a smaller standard deviation (0.17 compared with 0.24 for all women), but to provide accurate risks of having an affected pregnancy it is necessary to use the standard deviation based on all women.

When a screening marker has different standard deviations in affected and unaffected pregnancies risk reversal will occur, and if they are markedly different it will occur within non-extreme values of the marker.^4^ Accordingly, it is reasonable to set truncation limits near the point of risk reversal. Previously, a single lower truncation limit was suggested (0.65 MoM), but with the smaller standard deviation in unaffected pregnancies, it is necessary to have week-specific lower truncation limits to avoid the phenomenon of risk reversal. The previously reported upper truncation limit of 2.5 MoM from 10 to 13 weeks is still applicable.

The improvement in screening performance shown in Table 3 that arises from the smaller standard deviations of NT in unaffected pregnancies, while small is clinically useful – an approximate reduction in the false-positive rate of 20% or about four fewer false-positive per 1000 women screened without a reduction in the detection rate. The improvement in estimated screening performance was similar for the Combined and Integrated tests.
